# The Administration of Urethane

**Published:** 1886-04-20

**Authors:** 


**The Administration of Urethane.**—At a recent meeting of the Paris Societie de therapeutique ("Progr. med.," Feb. 6, 1886,) M. Huchard stated that the new hypnotic, urethane, given in doses of from 45 to 50 grains, dissolved in water, would produce a quiet sleep lasting six or eight hours. For children he recommended a solution of 3 grains of uerthane in 5 drachms of a mixture of orange-flower water, syrup, and distilled water—to be taken in the course of two days.

